# Rapid assessment of facilitators and barriers related to the acceptance, challenges and community perception of daily regimen for treating tuberculosis in India

**DOI:** 10.1080/16549716.2017.1290315

**Published:** 2017-05-09

**Authors:** Himanshu Negandhi, Ritika Tiwari, Anjali Sharma, Rajesh Nair, Sanjay Zodpey, Ramesh Reddy Allam, Ganesh Oruganti

**Affiliations:** ^a^ Indian Institute of Public Health – Delhi, Public Health Foundation of India, Gurgaon, India; ^b^ Public Health Foundation of India, Gurgaon, India; ^c^ SHARE India Research Office, Hyderabad, India

**Keywords:** Tuberculosis, RNTCP, DOTS strategy, daily regimen

## Abstract

**Introduction:** The Revised National Tuberculosis Control Program (RNTCP) is the largest tuberculosis (TB) control program in the world based on Directly Observed Treatment Short-Course (DOTS) strategy. Globally, most countries have been using a daily regimen and in India a shift towards a daily regimen for TB treatment has already begun. The daily strategy is known to improve program coverage along with compliance. Such strategic shifts have both management and operational implications. We undertook a rapid assessment to understand the facilitators and barriers in adopting the daily regimen for TB treatment in three Indian states.

**Methods:** In-depth interviews were planned across six districts of three purposively selected states of Maharashtra, Bihar and Sikkim, among health system personnel at various levels to identify their perspectives on adoption of a daily regimen for TB. These districts were sampled on the basis of TB notification rates. Thematic analysis of the qualitative data was undertaken.

**Results:** 62 respondents were interviewed from these 6 districts. During the analysis, it was observed that an easily accessible, patient-centred and personalized outreach is an enabling factor for adherence to treatment. Lack of transportation facilities, out-of-pocket expenses and loss of wages for accessing DOTS at institutions are major identified barriers for treatment adherence at individual level. At program level, lack of trained service providers, poor administration of treatment protocols and inadequate supervision by health care providers and program managers are key factors that influence program outcomes.

**Conclusion:** A major observation that emerged from the interviews is that the key to achieve a relapse-free cure is ensuring that a patient receives all doses of the prescribed treatment regimen. However, switching to a daily regimen makes adherence difficult and thus new strategies are needed for its implementation at patient and health provider levels. Most stakeholders appreciate the reasons for switching to a daily regimen. The stakeholders recognised the efforts of the Ministry of Health & Family Welfare (MoHFW) in spearheading the program. Strategies like the 99 DOTS call-centre approach may also further ensure treatment adherence.

## Background

The Revised National Tuberculosis Control Program (RNTCP) is the state-run tuberculosis (TB) control initiative of the Government of India. RNTCP is the largest TB control program in the world, placing more than 100,000 patients on treatment every month [1]. In 2015, a total of 9,132,306 TB suspects were examined by sputum smear microscopy and 1,423,181 cases were registered for treatment [2]. The disease expends a huge economic impact, with the estimated loss from TB in India amounting to a staggering 23.7 billion U.S.D in 2006 [3]. Launched in 1997, the RNTCP is based on the World Health Organization (WHO)-advised Directly Observed Treatment Short-Course (DOTS) strategy. The treatment consists of a shorter intensive phase of treatment with multiple drugs followed by a longer continuation phase with fewer drugs. The program’s success is defined as achievement of at least an 85% cure rate in new sputum positive pulmonary TB patients and detection of at least 70% of estimated cases. The improvement in program outcomes has been remarkable with rapid progress witnessed throughout the country. In India the program is well supported by proactive political commitment, improved funding, regular monitoring, quality drugs and direct observation. Although DOTS has been in existence in India for close to two decades, TB continues to be a leading cause of morbidity as well as mortality in India [4]. With an estimated 2.2 million new cases, India carries the highest burden of TB in the world. A staggering 220,000 deaths are reported annually. More than 110,000 people are co-infected with HIV/AIDS and TB every year [4]. The country has also witnessed a change in the disease epidemiology, with the emergence of higher rates of drug-resistant TB, a rising burden of TB among persons infected with HIV and the persistence of poor social determinants that put higher numbers of people at risk of acquiring TB infection.

The WHO in 2007, and again in 2010, advised daily treatment as the preferred drug regimen in treatment for all patients with TB. The WHO recommendations issued in 2010 highlight the importance of daily dosing of drugs for new patients in the intensive phase by stating ‘Wherever feasible, the optimal dosing frequency for new patients with pulmonary TB is daily throughout the course of therapy’. The guidelines emphasize that a daily dosing frequency, in both the intensive and continuation phases, is the optimal strategy for TB control: ‘Data shows that resistance to a single first line drug (isoniazid, ethambutol, or streptomycin) drives the development of MDR disease. National data on drug resistance has shown consistently high resistance to isoniazid, with rates of up to 40% in new patients’ [5]. A daily regimen is simple for the patient to understand, remember and adhere to until the completion of treatment [6]. However, even if a daily regimen has the advantage of simplicity in terms of patient understanding, the operational roll-out of a daily medication strategy can be quite challenging. Depending on the nature of supervision, a very intensive directly observed program strategy for a daily regimen could possibly increase the workload for health workers and undermine the full implementation of fundamental DOTS [7]. For patients, a daily regimen can adversely affect compliance to medication if patients have to sacrifice important family responsibilities and income [7]. Reasons for patients to default on medication are travelling back to their village, not being able to forgo their businesses for medication attendance, living far away and medication side-effects [7]. Both treatment and good socio-economic support given to the patients act as enablers for better adherence [8]. Although it is now believed that daily treatment can have equally high compliance rates, with the added benefit of less harm that may accrue from a single missed dose compared with the thrice-weekly regimen, DOTS for seven days a week presents a logistical challenge [9]. Mere DOTS without specific efforts such as transport coupons or food rations does not increase compliance [9]. A publication by Bose et al. in the Cochrane Database of Systematic Reviews compared the efﬁcacy and safety of intermittent, short-course anti-TB regimens (twice- or thrice-weekly) with daily short-course anti-TB regimens in treating childhood TB [10]. This review noted that parents of children should be educated regarding the need for adherence and treatment completion and appropriately compensated for trial-related expenses, to ensure continued participation. Proper follow-up needs to be done at regular intervals.

Studies done in several parts of the country show that relapses are closer to 10% or more in patients who received intermittent treatment compared with 5% among patients who received drugs daily [11]. Therefore the decision to continue with an intermittent therapy for TB control was considered inadequate to address the complex challenges posed by the disease. A decision to shift to the daily regimen has been taken by the Ministry of Health and Family Welfare (MoHFW). However, such a shift from an intermittent to a daily regimen has wide-ranging ramifications for public health systems. Against this background, it was recognized that before such an implementation, it would be necessary to understand the barriers and facilitators within the health systems for undertaking this shift.

## Methods

Our study was approved by the Institutional Ethics Committee (IEC) of the Indian Institute for Public Health – Delhi. The present study used primary data from interviews of key stakeholders.

**Figure 1.**
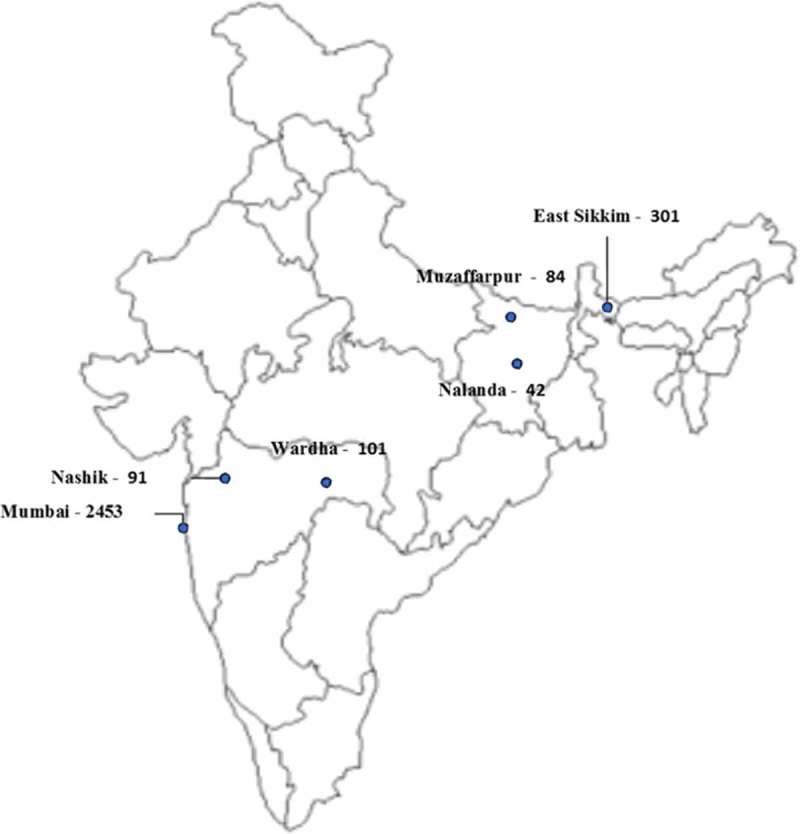
Location of districts covered under the study.

We undertook in-depth interviews (IDIs) across three purposively selected states of the country (Maharashtra, Bihar and Sikkim) from the five states that were identified by the Ministry of Health, Government of India for the roll-out of the daily regimen (Bihar, Himachal Pradesh, Kerala, Maharashtra and Sikkim). The location of these six districts on a map of India has been provided in Figure 1.

From these three states, we selected six districts for the IDIs (Table 1). We chose these districts on the basis of the case notification rate as reported in the TB India 2014 annual status report and in consultation with senior officials of the Central TB Division (CTD) [12].

**Table 1.** Selected districts and their case notification rates.

State	District	Case notification rate* `(per 100,000 population)
Maharashtra	Mumbai	2453 (Bail Bazar Road)
	Nashik	91
	Wardha	101
Bihar	Muzaffarpur	84
	Nalanda	42
Sikkim	East Sikkim	301

*Source: TB India 2014, annual status report.

We randomly selected Tuberculosis Units (TUs) in each of these districts. Each district was visited by a minimum of two research team members trained in qualitative data collection. The IDIs were conducted by administering semi-structured questionnaires to stakeholders that included program managers at the district level and health system personnel (Medical Officers [MO], District TB officers, RNTCP staff, DOTS providers, health workers including Senior T.B Laboratory Supervisors [STLS], Senior Treatment Supervisors [STS], laboratory technicians [LT], Accredited Social Health Activists (ASHAs) and Auxiliary Nurse Midwives (ANMs) to identify their perspectives on adoption of a daily regimen for TB.

The attitudes and perceptions of the respondents were explored through these IDIs. These interviews were conducted between September and December 2015. The analysis further helped us to produce more knowledge about the working or failure of health programs or policies related to adoption of a daily regimen for treatment of TB across other countries. The IDIs elicited participant perspectives about the health condition of the population, status of TB in their district, RNCTP and its functioning, DOTS strategy, treatment success, detection and management of adverse reactions to anti-TB drugs, supply chain, the current and anticipated challenges in a daily regimen and the possible strategies that state officials are likely to adopt to address these challenges within their public health settings. The senior officials of the CTD were also interviewed regarding the current RNCTP strategy and anticipated challenges in implementation of a daily regimen. The tools used for conducting IDIs with senior RNTCP officials, state/district program managers and M.Os and DOTS providers have been annexed in Appendices 1, 2 and 3.

**Figure 2.**
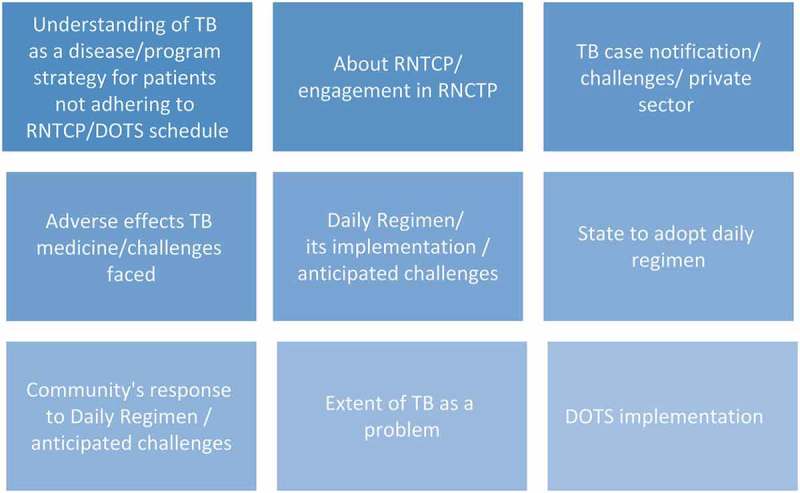
Identified themes for analysis.

The interviews were recorded using a voice recorder and data audio files were labeled, transcribed and translated (wherever applicable) into English and typed into MS *Word* files for further analysis. Each transcript was reviewed by study team members for completeness and appropriateness. We then undertook a thematic data analysis focusing on examining themes within the data. The responses were suitably placed for analysis under the themes set out in Figure 2.

## Results

### IDIs

#### Understanding of TB

The knowledge level of respondents related to the aetiology and epidemiology of TB was found to be satisfactory. When asked to describe TB, its mode and spread of infection, respondents across the district and sub-district levels showed adequate knowledge about the disease. DOTS providers were aware of the types of TB (pulmonary and extra-pulmonary TB), treatment regimen, and sources of exposure and risk of infection:

TB disease is caused by the organism mycobacterium tuberculosis… and is spread by when the patient coughs… more than 15 patients are infected. (ASHA, Maharashtra)

TB is an infectious disease and it spreads from one person to another person. It spreads through coughing, sneezing and spitting here and there… while coughing they should place the handkerchief around the mouth and should not cough near others. If cough persists…we bring them to Primary Health Centre (PHC) for testing. Treatment is for 6 months. (ASHA, Bihar)

It is spread through cough, through air. Its symptoms are continuous cough for two weeks, fever at night, loss of appetite and weight loss. These are the four symptoms which indicate that TB can be there. If cough persists for two weeks, then sputum should be tested at your nearest health facility i.e. govt. health facility where it is for free. (Senior DOTS Plus/HIV TB supervisor, Maharashtra)

The knowledge and awareness of STS and STLS on TB, its mode of spread and infectiousness were sound across all the interviews.

#### Engagement in RNCTP

Our study respondents had a long experience of having worked in the national program for controlling TB. Critical insights were shared on the current RNTCP framework and challenges being faced in the program’s implementation. Although dedicated work responsibilities for TB activities have the potential to provide intensive supervision, an existing workforce shortage among senior staff at the district level has necessitated taking on some additional responsibilities of other national health programs. However, this was not a uniform experience across all states:

At present I have only RNTCP as my responsibility at district level. (Medical Officer-Tuberculosis Control (MOTC), Sikkim)

Similarly the officials at TU level such as MO-TC, Senior Treatment Supervisors (STS) and STLS have reported having only RNTCP activities as their key responsibilities. However, the MO i/c and lab technicians at Designated Microscopy Centre level, DOTS providers, health workers (Front Line Workers (FLWs) like ASHA, ANM, General Nursing & Midwife (GNM)) etc. responded that they had additional responsibilities (assigned to them from time to time) other than the activities related to the RNTCP program.

#### Notification of TB

TB case notifications from private sector care providers in India rose between 2013 and 2014 [13]. Many centers were making additional efforts to increase the notification rate and striving for better coordination between the public and the private sectors. The role for private providers has been prominently identified under RNTCP as effectively reaching out to the un-reached. As the disease notification has been made mandatory, the private providers who treat TB cases are being reached out to by the health workers, motivating them to refer new cases or notify such cases:

To get the notification we have conducted a CME [Continued Medical Education], with the help of I.M.A [Indian Medical Association]. We are sending our workers every month to the private practitioners and ask them how many patients they have, from which place they have come and what treatment they are giving them. We collect the data in the formats and enter them in NIKSHAY. (Additional District Health Officer in-charge, Maharashtra)

Mostly doctors notify because they don’t want to get to any problem. The only problem is about the seriousness of time line. (District TB Officer (DTO), Maharashtra)

Smart reporting options with a technology-based interface with the private practitioners have also been introduced from the central government recently such as the option of registration and login for private facilities for TB notification indirectly in NIKSHAY software and call center facility. However, complete notification from all the private sectors has still not been attained.

#### Understanding adverse effects of TB medicines

The commonly encountered adverse effects on intermittent therapy were nausea, headache, loss of appetite, colored urination, joint pain, loss of vision, hearing problems and jaundice. Almost all of the respondents mentioned that the patients with such symptoms inform health workers and DOTS providers, who in turn may report these events to their MO in charge. Such cases are usually referred to the doctors for further counseling and treatment.

Regarding management of the adverse events by the medical doctors, although they expressed confidence in their own abilities to manage such cases, they lamented about the absence of basic drugs to manage acidity and gastritis in the treatment boxes:

Side-effects are well managed because we have doctors in hospitals and PHCs. With these drugs there is problem of acidity and gastritis. But we are not given antacids in the beginning in the box because patients have the problem even with the basic drugs which they take for the first time. Then the RNTCP staff has to refer them to the hospital for such small things. Then we lose the patient. (City TB officer, Maharashtra)

The absence of a robust adverse effects monitoring system was highlighted and a point of concern for respondents. Doctors, especially those in managerial positions at the state and the central levels, were cognizant of its importance.

There were divided views on whether adverse reactions would be higher on an intermittent regimen or a daily regimen. Since an intermittent regimen has high drug dosage to account for the intermittent nature of drug administration, it is expected to show high levels of gastro-intestinal intolerability. Daily regimens on the other hand are expected to have higher biochemical abnormalities (liver function).

##### Roll-out of daily regimen

A major observation that emerged from the interviews was that key to achieving a relapse-free cure is to receive all doses of the treatment regimen as prescribed. However, switching to a daily regimen makes adherence difficult and thus new strategies are needed for its implementation at patient and health provider levels. Most of the responses pertinent to roll-out of the daily regimen were supportive.

The concept of ‘family DOTS’ in the daily regimen was suggested as one way to address the issue of patients coming to the health centre daily. Most health workers suggested that DOTS should be provided by any identified/responsible family member such as spouse, parent/s, sibling etc. to ensure better adherence. However, it was suggested that an ASHA worker will remain as a prime motivator and provider to the patient and to the identified family member. STS and TB volunteers can further supervise the ‘family DOTS’ with improved outreach and supervision plans:

Once the daily regimen comes the same problem will be there that the working people won’t be able to attend the health facility daily so the DOTS providers, strong DOTS provider have to be identified who will give them daily medicines. And I think one day directly observed from the hospital and the six days to the family or the DOTS provider should be given… If I am a family member and one of my family members has TB, I will be more aware and more concerned that he becomes all right. (M.O, Nashik)

However, the MoHFW is already trying to use science and technology to ensure adherence through a direct regimen. Strategies like the 99 DOTS call-centre approach may also further ensure treatment adherence:

In daily regimen patient will be under observation because they are having the strip of 28 days… we have to make analysis… We can use to information technology for adherence system… We are definitely sorting out that particular question, like giving the alternative mechanism by daily regimen, by 99 DOTS. (Senior official, CTD)

…In future we want to start missed calls to call centres… it will be incorporated in the Nikshay itself… That is the future plan. (Senior Official, CTD)

Challenges related to storage space for drugs, availability of the reagents and containers were not expressed.

##### Shift to daily regimen and anticipated challenges

The need to shift from the current intermittent DOTS to a daily regimen has been felt due to the rationale that there is a high rate of relapse under the intermittent regimen, and an increase in drug-resistant TB:

Daily dosage will help. We will have less number of defaulters and acceptability will be good. It was difficult for us to explain to the patient that why it is alternate days here and why it is daily dosage in the private. Educated people opt for private. (D.T.O, Maharashtra)

Daily regimen will be more useful. Because of this alternate dose is also very high. In one blister seven tablets are there. In daily it will be reduced. (LT, Bihar)

When asked to state a preference for fixed dose combination (FDC) or loose drugs, choice of loose drugs was put forth. The need for the program to be better integrated with the general health system to address the challenge of shortage of human resources at the TU level was also deliberated.

The workload implications of a daily DOTS were perceived to be a challenge:

Even if the TB health visitor [TB.H.V] is absent they give DOTS, but the problem will be with daily regimen. They are busy with their program of dengue, malaria, immunization program. So I don’t know how much time I will be able to give for DOTS. Problem will crop up when their TBHVs remain absent… (DTO, Maharashtra)

##### Community’s perspective towards daily regimen

When asked about the community perspective on the use of a daily regimen vs intermittent regimen, the majority of respondents highlighted that the community would definitely prefer the daily regimen over the intermittent regimen considering the likelihood of reduced dosages, parity with the private providers’ treatment choice and lesser adverse effects expected from the revised TB treatment using the daily regimen. The role of counselling was also emphasized in bringing a smooth transition from the intermittent to daily regimen and thus ensuring fewer drop-outs and more adherence:

There won’t be reluctance. Program has been there, so we have to explain them that govt. has brought daily DOTS at par with the private. Patient acceptance and tolerance will be more. Side-effects will be less. But one should be active in explaining like group counseling etc. (DTO, Mumbai)

#### Demand for quality TB services

The health providers are expected to provide adequate information about TB to the patients. The RNTCP aims to ensure universal access to quality-assured TB care as per the Standards for TB Care in India (STCI). The RNTCP’s other initiatives for assurance of quality services include: quality case management, notification of all TB cases in Nikshay, access to quality TB diagnosis for pediatric cases through Cartridge-based Nucleic Acid Amplification Testlabs etc. Since chemotherapy with anti-TB drugs is the mainstay of the RNTCP, the program will have to be especially vigilant about the quality of the supplied drugs. Such a perception was also echoed in our interviews where participants felt that the program needs to invest sufficient resources in procuring and supplying quality anti-TB drugs in the treatment boxes:

First of all, the quality and potency of drugs should be maintained. So, that it is effective. TB is a serious problem in our state and country. It is a communicable disease. So, the patients should be provided with good-quality drugs. (Treatment Organizer, Sikkim)

They [TB patients] are unaware of the quality of the drugs. They trust the government that they are getting good treatment and will get cure. (Treatment Organizer, Sikkim)

### Universal coverage and sustainability

The RNTCP’s theme for the 12th Five Year Plan is to provide universal access to quality diagnosis and treatment for all TB patients in the community. The RNTCP’s vision is to significantly reduce the TB burden in India by ensuring universal access to quality-assured TB care as per the STCI [12]:

This is not a question of coverage or not coverage. We want to do it [daily regimen] for entire country. (Senior official, CTD)

I see the strength as its [RNTCP’s] reach… since it is being implemented by state health services so they have a reach… But the other side is they have covered all the country, but it is yet to actually deliver the services. There are issues with accessibility because of the stringent guidelines which the program has. This transition to daily regimen, we are also looking it as an opportunity to make the program much more flexible and to look into other avenues in which it is much more patient friendly. (Senior Official, CTD)

It has been understood that decentralization of inventory management practices is very important for long-term sustainability of the program. There is a need for the states to manage their drug logistics as per RNTCP guidelines. Regular trainings and re-trainings on procurement and supply chain management are conducted by the CTD time and again [12]:

One committee is there, national committee and at CTD level also we are having a committee where we are supervising and coordinating the activity. (Senior Official, CTD)

## Discussion

The WHO recognizes in its STCI that all patients should be given a daily regimen under direct observation [14]. However, the country program may consider a daily or intermittent regimen for treatment of TB depending on the available resources and operational considerations [15]. The WHO emphasizes that a patient-centered approach, use of Information and communication technology (I.C.T), patient counseling and health education are vital for TB programs. Supervision and support also need to be individualized [15]. Published evidence is available on implementation of a daily regimen from multiple countries globally. When a patient-centered approach was adopted in Tanzania, a comparison of patients treated with such an approach versus patient treatment in a health facility-based DOTS showed similar cure rates and better treatment success rates in the patient-centered treatment approach [16]. However, even if a daily regimen has the advantage of simplicity in terms of patient understanding, the operational roll-out of a daily medication strategy can be quite challenging. Depending on the nature of supervision, a very intensive directly observed program strategy for a daily regimen could possibly increase the workload for health workers and undermine the full implementation of fundamental DOTS [7]. For patients, a daily regimen can adversely affect compliance to medication if patients have to sacrifice important family responsibilities and income [7]. Reasons for patients to default on medication are travelling back to their village, not being able to forgo their businesses for medication attendance, living far away and medication side-effects [7]. Experience from India suggests that both intermittent and daily regimens had comparable conversion rates at the end of the initial intensive phase of treatment for TB [17].

Patient adherence to therapy is of great importance in national programs for tackling TB. The disease has historically being associated with stigma, which affects the early access to treatment. A delay in the initiation of appropriate therapy is bound to lead to more extensive disease at the time of diagnosis; in such a scenario programs emphasize an early visit to a health center for diagnosing TB in the presence of suggestive symptoms of the disease. The presence of stigma with TB has been recognized, is a high-priority concern for program managers and has been highlighted by a USAID publication [18].

It is well known that there exists a large private sector (both formal and informal) in India. More than 80% of out-patient visits in the country are made to the private sector. It is therefore quite natural that the private sector would be the first point of contact for a high proportion of TB symptomatics. In such a scenario, a comprehensive TB control program needs to effectively partner with the private sector across the diagnosis, notification, treatment and follow-up of TB patients in the country. India has introduced app-based notifications that private providers can send to the RNTCP [12]. A similar approach for increasing TB notifications from the private sector has also been made by roping in call centers which can be dialed-in free of cost by private providers who intend to notify TB patients under their care. Such an effort is expected to increase the overall number of notified cases to the RNTCP, thereby presenting a correct picture of the actual TB burden in the community. Sairu Philip and colleagues have recognized that notification of TB as a public health measure for control of TB needs to be showcased to the private practitioners in India [19].

Private sector participation cannot just be limited to improving notification, it must also be sensitized to use the most appropriate diagnostic protocol and suggest the appropriate individualized care regimen for a patient. The RNCTP has to popularize, and insist upon private practitioners following, the STCI. This will need support from practicing doctors, as well as the Indian Medical Council for popularizing the STCI among its members.

When compared to adult patients of TB, there is greater evidence of the benefits of a daily regimen for TB among children. Menon et al. have noted that the default rates may be influenced by other factors (like overcrowding in the household, or default in the first month in children with TB) [20]. Twice-weekly intermittent short-course therapy is less likely to cure TB in children as compared to daily therapy [20]. A publication by Bose et al. in the Cochrane Database of Systematic Reviews compared the efﬁcacy and safety of intermittent, short-course anti-TB regimens (twice- or thrice-weekly) with daily short-course anti-TB regimens in treating childhood TB [10]. This review noted that parents of children should be educated regarding the need for adherence and treatment completion and appropriately compensated for trial-related expenses, to ensure continued participation. Proper follow-up needs to be done at regular intervals.

Thus, while the current evidence and global opinion point to superiority of the daily regimen in ensuring better TB control, there are recognized challenges in the delivery of such a regimen through the public health system. National programs will have to increasingly demonstrate meaningful partnerships with the private sector in order to ensure universal notification and a complete adherence to the recommended Standards of Care for TB. Additionally, national programs will have to evolve strategies that minimize stigma, encourage adherence, ensure regular monitoring and systematically expand quality health service provision across the country. A regimen change (from intermittent to daily) is expected to continue delivering such services.

Most of the respondents in our study were supportive towards roll-out of a daily regimen. Since an intermittent regimen has high drug dosage to account for the intermittent nature of drug administration, it is expected to show high levels of gastro-intestinal intolerability. Daily regimens on the other hand are expected to have higher biochemical abnormalities (liver function). CTDs can plan to devise a roll-out strategy for phasing in the daily regimen across the country.

However, advocacy for the superiority of the daily regimen should be mainstreamed through scale-up of advocacy, communication and social mobilization (ACSM) strategy. Development and dissemination of revised operational guidelines for functionaries at all levels are necessary. A training plan for all levels of the health system should be prepared and implemented at the earliest opportunity. The RNTCP should design an easy but comprehensive format for monitoring the roll-out of the daily regimen and for monitoring adverse effects of drugs. Stock monitoring using ICT, logistics and supply chain management should also be undertaken appropriately.

For improving patient adherence to treatment, the RNTCP can provide a travel pass (by roadways or train) for the patients to visit a DOTS center during the course of treatment [21]. Nutritional supplementation for TB patients through existing government schemes like the Public Distribution System should be encouraged [21]. The program may evaluate the possibility of using a family member as a DOTS provider. For increased patient adherence, I.C.T-based patient reminder systems need to be implemented [22].

The main strength of our study is that we developed our protocol through a consultative process by engaging and soliciting feedback from senior officials of the CTD. Our sampling was based on RNTCP performance criteria as agreed upon with multiple stakeholders. Out of five states identified for roll-out of the daily regimen, three states were included in our study. The selection of states was done based on epidemiological information (from the Annual TB Report, 2014) and experts’ opinion. Our research team had considerable experience in undertaking IDIs with health system personnel. All team members who were responsible for taking interviews were provided with refresher training before the onset of data collection. We obtained perspectives across all levels of the health system. We interviewed grassroots workers, district and state-level program personnel, as well as representatives from the CTD. We had a sizable number of respondents across various categories of respondents.

We were limited in obtaining the perspectives of stakeholders within the public sector. Although we recognized that private sector participation is both vital as well as helpful in the control of TB, the limited time precluded any exploration of their perspectives. Since most of our participants had considerable experience of working with the RNTCP, this may have affected their responses in the interviews. The limitations of our study include that we could not answer the question as to how do we ensure directly observed therapy (DOTS) when we switch from thrice-weekly to daily therapy? The key to achieving a relapse-free cure is to receive all doses of the treatment regimen as prescribed. A shift to daily therapy makes it difficult and thus new strategies for DOTS or additional increase in labor (i.e. observation daily) and additional burden on patients (i.e. needing to come in daily for dosing) are needed. Our interviews provided limited insights into how we should ensure and monitor treatment adherence.

## Conclusions

A daily regimen for TB is globally accepted for disease control. In India, such a shift from an intermittent to a daily regimen has wide ramifications for public health systems. Such a strategic shift from intermittent therapy to a daily regimen will have both management and operational implications with regard to the development of revised treatment protocols and guidelines, procurement of medicines, managing clinical manifestations, addressing the need for training based on revised protocols and revising monitoring and evaluation indicators.

There is a need for strengthening operational guidelines and RNTCP formats for smooth roll-out of the daily regimen at national level. The program must strictly monitor adequate and timely release of funds, and payments of salaries/incentives for all personnel involved in the program. Enhanced engagement with the private sector for TB control is also needed. Scale-up of existing support for notification of TB cases from the private sector can be achieved by leveraging technology in the form of apps/call centers/WhatsApp etc. CME sessions can also be conducted through associations like the IMA and others for sensitizing private providers to the STCI and daily regimen.

## Paper context

The Revised National Tuberculosis Control Program (RNTCP) is the largest tuberculosis (TB) control program in the world based on Directly Observed Treatment Short-Course (DOTS) strategy. Globally, most countries have been using a daily regimen and in India a shift towards a daily regimen for TB treatment has already begun. We undertook a rapid assessment to understand the facilitators and barriers in adopting a daily regimen for TB treatment in three Indian states.

## Appendix 1

Tool 1: In-Depth Interview (I.D.I) Questionnaire – Senior Officials of RNTCP

Performa No.:

***Questionnaire***

Respondent’s details

Since when are you engaged with RNTCP?

Can you tell us your current responsibilities in RNTCP?

1.3 Do you have any additional responsibilities in other disease control programs?

Respondent’s perspectives

What are your thoughts about RNTCP?

*[Probes: program design, program formats, program indicators, operational guidelines, reporting systems, feedback mechanism, supportive supervision, availability of dedicated budget, trainings, overall performance, current intermittent dosage strategy, adverse effects, supply chain, storage facility, procurement, DOTS strategy, MDR–TB, XDR–TB, disease notification, role of private providers, HIV–TB co-infection]*

*What is the process of TB case notification in your work setting?*

*How does RNTCP obtain the data for its annual TB report in the public sector? What are the challenges?*

*[Probes: challenges]*

*How does RNTCP obtain the data for its annual TB report in the private sector? What are the challenges?*

*[Probes: challenges]*

*Do you anticipate adverse effects due to TB medicine?*

*[Probes: if yes, then how are they reported? Who assess them? Is there a register?]*

What are your thoughts about daily regimen for TB?

*[Probes: which is more beneficial and why?]*

What (additional/relieving) responsibilities do you envisage if a daily regimen for TB is implemented at national/state/district level?

What challenges do you anticipate if we shift from intermittent to daily regimen?

*[Probes: manpower, logistics and supplies (procurement), side-effects, supervision and monitoring, cost, incentive to patients on treatment completion, motivation through outreach, DOTS non-adherence of treatment/default, training, Management Information System (M.I.S), performance indicators, HIV–TB co-infection.]*

In your opinion, how will the community respond to shifting to the daily regimen?

*[Probes: acceptance of people/views about intermittent therapy, views about current treatment regimens]*

How will you address these challenges at your level?

How can the states/districts address these challenges?

How can we choose state/district who should adopt a daily regimen?

*[Probes: criteria, will it only be RNTCP-related or include other national health programs?]*

Do you wish to provide any additional information related to the study?

Thank you for your participation.

If we require any additional information, is it alright if we contact you over the phone on a day and time convenient to you?

Any specific observations/information related to the interview:

## Appendix 2

Tool 2: In-Depth Interview (I.D.I) Questionnaire – State/District Program Managers and Medical Officers

Performa No.:

***Questionnaire***

Could you tell us/me about your experience as a medical officer?

*[Probes: exact title of current position, no. of years of work experience, place of work (current position) – PHC/Community Health Centre/Sub-district/Sub-divisional Hospital /District Hospital/State level, common diseases prevalent in the community]*

Do you have any additional responsibilities in other disease control program?

Can you tell us about the extent of the TB problem in your community?

What are your thoughts about RNTCP?

*[Probes: program design, program formats, program indicators, operational guidelines, reporting systems, feedback mechanism, supportive supervision, availability of dedicated budget, trainings, overall performance, current intermittent dosage strategy, adverse effects, supply chain, storage facilities, procurement, DOTS strategy, M.D.R–TB, X.D.R–TB, disease notification, role of private providers, HIV–TB co-infection]*

*How is the program functioning in your State/District?*

*[Probes: morbidity and mortality due to TB? Rough estimate of new cases every month, profile of the patients, T.U statistics, STS, S.T.L.S, are the funds released on time?, B.C.G coverage]*

Since when are you implementing DOTS in your area?

What is the process of TB case notification in your work setting?

How does RNTCP obtain the data for its annual TB report in the public sector? What are the challenges? *[Probes*: *challenges* *]*

How does RNTCP obtain the data for its annual TB report in the private sector? What are the challenges? *[Probes*: *challenges* *]*

Do you anticipate adverse effects due to TB medicine?

*[Probes: if yes, then how are they reported? Who assess them? Is there a register?]*

What are the challenges you face while treating TB patients?

*[Probes: M.D.R–TB? Adherence of the patient in completion of DOTS? Cost? Side-effects? Social stigma? Superstitions prevalent in the community? Transfer-in/transfer-out/migration? Any other challenges?]*

What are your thoughts about a daily regimen for TB?

In your opinion, what are the possible obstacles in implementation of daily regimen therapy?

*[Probes: manpower, logistics and supplies (procurement), side-effects, supervision and monitoring, cost, incentive to patients on treatment completion, motivation through outreach, non-adherence of treatment/default, training, M.I.S, performance indicators.]*

How will you address these challenges at your level?

How can we choose state/district who should adopt a daily regimen?

*[Probes: criteria, will it only be RNTCP-related or include other national health programs?]*

In your opinion, how will the community respond to shifting to the daily regimen?

*[Probes: acceptance of people/views about intermittent therapy, views about current treatment regimens]*

Do wish to provide any additional information related to the study?

Thank you for your participation.

If we require any additional information, is it alright if we contact you over the phone on a day and time convenient to you?

Any specific observations/information related to the interview:

## Appendix 3

Tool 3: In-Depth Interview (I.D.I) Questionnaire – DOTS providers – Health Workers (F.L.Ws like ASHA, ANM, GNM)

Performa No.:

***Questionnaire***

Could you tell us your understanding on TB?

*[Probes: awareness about the disease, spread of infection, symptoms, duration and availability of treatment, cure]*

What is the program strategy for patients who are not adhering to RNTCP/DOTS schedule?

*[Probes: how do you follow up, no. of home visits, review of defaulters, reporting to the M.O/Designated Microscopy Centres?]*

Are you facing any difficulties in the current RNTCP program strategy?

*[Probes: adherence, non-compliance, default, testing, unavailability of the drug, supply chain, storage facilities, incentives on treatment completion, availability and access of DOTS provider, transportation]*

Do private providers/doctors/clinics notify TB?

What is the role of the private health care providers in your area?

*[Probes: coverage, rapport with the community, community’s attitude for seeking treatment from them, mutual relations with DOTS providers]*

How do you detect adverse effects due to TB medicines?

*[Probes: register, about district level]*

If we start a daily regimen for TB, in your opinion, do you think it will be useful or not? And why?

*[Probes: challenges, how does it influence your workload, outreach, supply chain issues, incentive to patients on treatment completion, non-adherence of treatment/default, district preparedness for the implementation of daily regimen if any? What will be your new activities/role in managing TB cases in your community?]*

In your opinion, what is the perception and acceptance of the community regarding DOTS?

In your opinion, how will the community respond to shifting to the daily regimen?

*[Probes: acceptance of people/views about intermittent therapy, views about current treatment regimens]*

Do wish to provide any additional information related to the study?

Thank you for your participation.

If we require any additional information, is it alright if we contact you over the phone on a day and time convenient to you?

Any specific observations/information related to the interview:
